# Oncogenic B-Myb Is Associated With Deregulation of the DREAM-Mediated Cell Cycle Gene Expression Program in High Grade Serous Ovarian Carcinoma Clinical Tumor Samples

**DOI:** 10.3389/fonc.2021.637193

**Published:** 2021-03-04

**Authors:** Audra N. Iness, Lisa Rubinsak, Steven J. Meas, Jessica Chaoul, Sadia Sayeed, Raghavendra Pillappa, Sarah M. Temkin, Mikhail G. Dozmorov, Larisa Litovchick

**Affiliations:** ^1^Division of Hematology, Oncology and Palliative Care, Department of Internal Medicine, Virginia Commonwealth University, Richmond, VA, United States; ^2^Division of Gynecologic Oncology, Department of Obstetrics and Gynecology, Virginia Commonwealth University, Richmond, VA, United States; ^3^School of Medicine, Virginia Commonwealth University, Richmond, VA, United States; ^4^Department of Pathology, Virginia Commonwealth University, Richmond, VA, United States; ^5^Massey Cancer Center, Virginia Commonwealth University, Richmond, VA, United States; ^6^Department of Biostatistics, Virginia Commonwealth University, Richmond, VA, United States; ^7^Department of Pathology, Virginia Commonwealth University, Richmond, VA, United States

**Keywords:** MYBL2, DYRK1A, cancer genome atlas, protein complex, transcription, FoxM1, LIN9

## Abstract

Cell cycle control drives cancer progression and treatment response in high grade serous ovarian carcinoma (HGSOC). *MYBL2* (encoding B-Myb), an oncogene with prognostic significance in several cancers, is highly expressed in most HGSOC cases; however, the clinical significance of B-Myb in this disease has not been well-characterized. B-Myb is associated with cell proliferation through formation of the MMB (Myb and MuvB core) protein complex required for transcription of mitotic genes. High B-Myb expression disrupts the formation of another transcriptional cell cycle regulatory complex involving the MuvB core, DREAM (DP, RB-like, E2F, and MuvB), in human cell lines. DREAM coordinates cell cycle dependent gene expression by repressing over 800 cell cycle genes in G0/G1. Here, we take a bioinformatics approach to further evaluate the effect of B-Myb expression on DREAM target genes in HGSOC and validate our cellular model with clinical specimens. We show that *MYBL2* is highly expressed in HGSOC and correlates with expression of DREAM and MMB target genes in both The Cancer Genome Atlas (TCGA) as well as independent analyses of HGSOC primary tumors (*N* = 52). High B-Myb expression was also associated with poor overall survival in the TCGA cohort and analysis by a DREAM target gene expression signature yielded a negative impact on survival. Together, our data support the conclusion that high expression of *MYBL2* is associated with deregulation of DREAM/MMB-mediated cell cycle gene expression programs in HGSOC and may serve as a prognostic factor independent of its cell cycle role. This provides rationale for further, larger scale studies aimed to determine the clinical predictive value of the B-Myb gene expression signature for treatment response as well as patient outcomes.

## Introduction

High grade serous ovarian cancer (HGSOC) is the most common subtype of ovarian cancer (1). Scientific understanding of this disease is a priority as ovarian cancer remains the most lethal of the gynecologic malignancies (2). Better understanding of the factors that contribute to the pathogenesis and progression of HGSOC is required for improving the diagnostics and treatment of this disease. B-Myb (encoded by *MYBL2*) is a transcription factor oncoprotein that contributes to cell proliferation and poor clinical outcomes in cancer (3). B-Myb is recognized as a prognostic indicator in breast cancer and is included within validated scoring systems commonly used to assess the risk of disease recurrence (4, 5). Interestingly, *MYBL2* gene copy-number gain is present in 55% of HGSOC cases in both The Cancer Genome Atlas (TCGA) as well as data set; however, the prognostic importance of B-Myb in this disease has not been well-characterized (6). Previous *in vitro* studies of cancer cell models found that high B-Myb expression not only deregulates the cell cycle through MMB formation and subsequent expression of genes required for mitosis, but leads to disruption of the repressor complex DREAM (DP, RB-like, E2F, and MuvB), a master regulator of the cell cycle dependent gene expression (7).

DREAM assembles when RB-like protein p130 binds to MuvB protein complex containing LIN52, LIN9, LIN37, LIN54, and RBBP4, and mediates global repression of both early and late cell cycle genes in G0 and G1 (8). Upon cell cycle re-entry, DREAM dissociates in a cyclin D-CDK4/6-dependent manner, resulting in transcription of genes required for coordinated cell cycle progression, including B-Myb (9). MuvB then binds to B-Myb in the S phase to initiate the expression of late cell cycle genes, which peaks in the G2/M phases upon recruitment of FoxM1 transcription factor to their promoters in B-Myb and MuvB-dependent manner (10). Therefore, by forming 3 distinct transcriptional regulatory protein complexes, MuvB ensures proper expression of cell cycle genes throughout all phases of the cycle (11).

We previously determined that 49 of the 50 most differentially expressed genes in the HGSOC TCGA dataset with high B-Myb expression were validated DREAM target genes. Furthermore, genes encoding the MuvB subunits were altered in the majority of HGSOC cases, both by gene copy number losses (LIN52, LIN54) and gains (LIN9, LIN37). Together, this suggests that DREAM functional status may carry prognostic implications. Indeed, DREAM maintains cellular dormancy and has been implicated in HGSOC spheroid formation as well as treatment resistance in human cancer cells derived from the ascitic fluid (12). The DREAM complex may also have secondary effects, aside from its predominate cell cycle role, through involvement in the DNA damage response and by regulating the expression of genes involved in homologous recombination, such as *BRCA1/2* and *RAD51* (13).

To further investigate the roles and relationships between B-Myb, DREAM, and MMB in HGSOC, we sought to characterize the expression and prognostication of B-Myb in HGSOC. We additionally aimed to corroborate the molecular model of B-Myb-mediated DREAM complex disruption through biostatistical analyses of previously validated data sets and gene expression studies of patient-derived HGSOC tumor samples. Finally, we provide the rationale for evaluation of DREAM functional status in HGSOC and how B-Myb expression may serve as a potential surrogate marker for DREAM assembly. Our ultimate goal is to contribute to the ongoing development of predictive transcriptional signatures for treatment response and disease progression in HGSOC.

## Materials and Methods

### Quantitative PCR

RNA was isolated using MagMAX^TM^ kit (ThermoFisher) and used to synthesize cDNA using SensiFAST^TM^ kit (Bioline). qPCR with Maxima SYBR Green/ROX master mix (ThermoFisher) and gene specific primers was performed using Applied Biosystems 7900HT. Fold changes in mRNA expression relative to controls were calculated using the 2ΔΔCt methodology.

**Table:** 

**Primers**	**Sequences (5'-3')**
LIN52	Forward: TCACGTGACATGGGTTGGAA
	Reverse: TCCAGATCTGTCCCGTCTGT
18S rRNA	Forward: AACCCGTTGAACCCCATT
	Reverse: CCATCCAATCGGTAGTAGCG
FOXM1	Forward: GTCTGGAGGGTCCACACTTG
	Reverse: CGACGGGGGCTAGTTTTCAT
MYBL2	Forward: CATTGTGGATGAGGATGTGAAGC
	Reverse: TGGTTGAGCAAGCTGTTGTCTTC
CCNB2	Forward: GCTCCAAAGGGTCCTTCTCC
	Reverse: TGCAGAGCAAGGCATCAGAA
AURKA	Forward: TGGCAAATGCCCTGTCTTACTGTCA
	Reverse: GGGGGCAGGTAGTCCAGGGT
LIN9	Forward: ATTCGGCGGCTTATGGGAAA
	Reverse: AGAGCCTTATTTTCTGCCGT
KIF23	Forward: TGCTGCCATGAAGTCAGCGAGAG
	Reverse: CCAGTGGGCGCACCCTACAG
E2F1	Forward: GCCACTGACTCTGCCACCATAG
	Reverse: CTGCCCATCCGGGACAAC
DYRK1A	Forward: ACACCAATTTCCGAGGGGTC
	Reverse: AAGGCATTCCCAGTAGCACC
PCNA	Forward: GATAACGCGGATACCTTGGC
	Reverse: CTCCGTCTTTTGCACAGGAAA
MCM2	Forward: GGCGGAATCATCGGAATCCT
	Reverse: ATCATCCAGAGCCAGTCCCT

### Biostatistics

**Figure 1:**
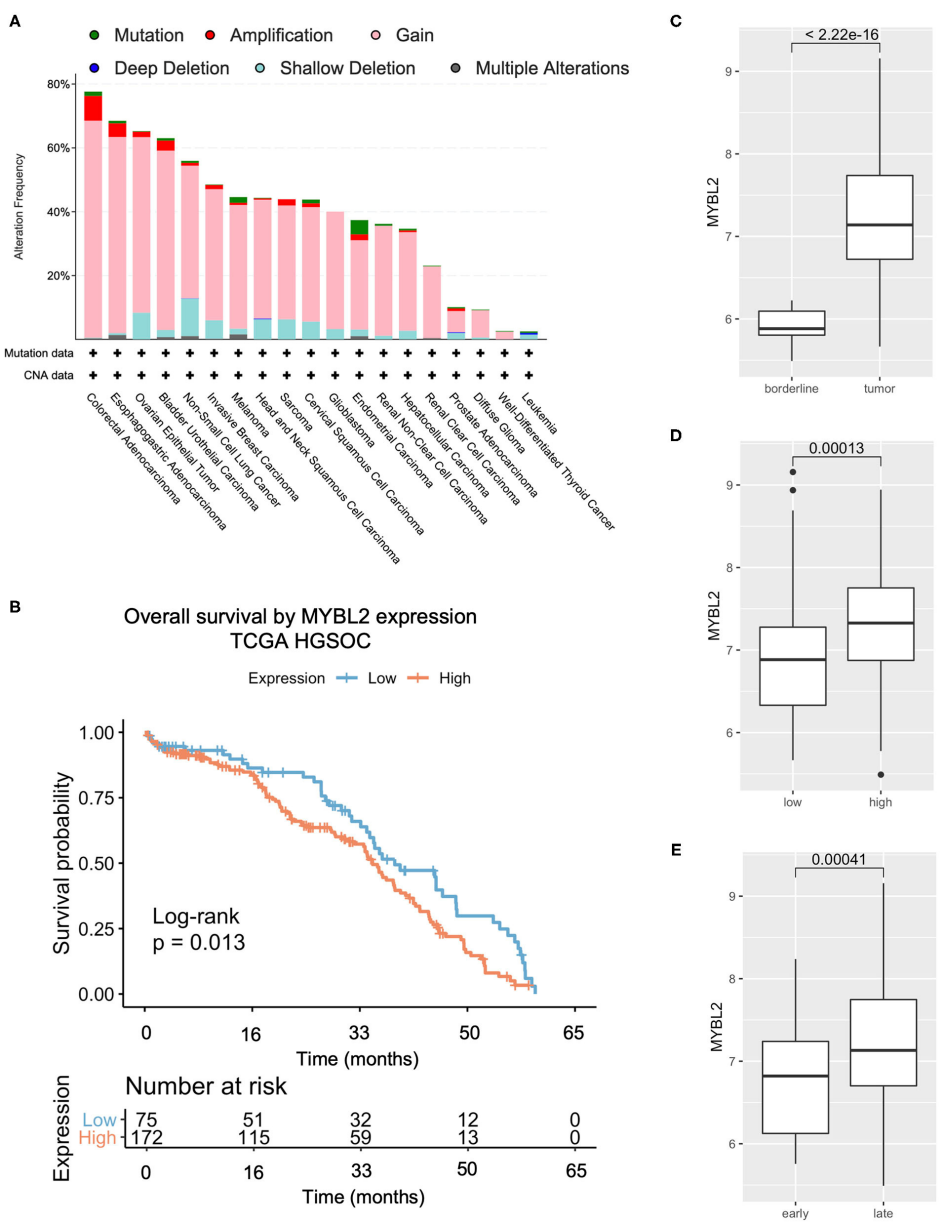
*MYBL2* expression is altered and associated with poor overall survival in HGSOC. **(A)** TCGA summary showing frequent (65%) alterations in *MYBL2* in HGSOC (*N* = 584), mostly gains (55%, *N* = 321 of 584 cases). **(B)** Kaplan–Meier survival analysis of TCGA HGSOC data of all tumor stages. **(C)** Published gene expression dataset GSE9891 was analyzed for expression of *MYBL2* in primary untreated ovarian carcinoma (*N* = 267) as compared with borderline ovarian surface epithelial-stromal tumor (*N* = 18) (16). Using data from the same study, expression of MYBL2 was compared between high (*N* = 163) and low (*N* = 116) grade ovarian tumors **(D)** as well as between early stage (*N* = 42) and late stage (*N* = 240) tumor samples **(E)**. Welch 2 Sample *t*-test, *p* <0.01 for **(C–E)**.

**Figure 2:**
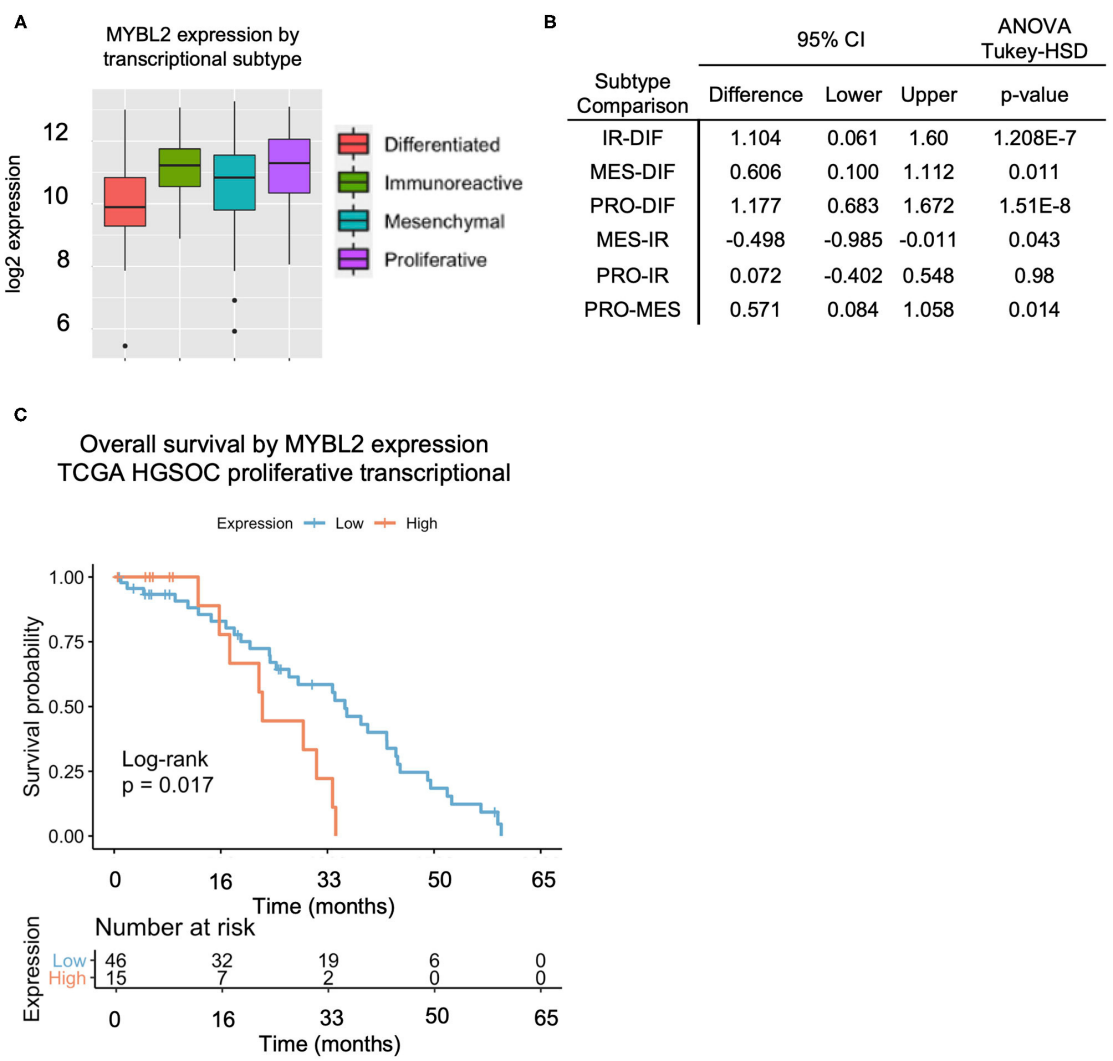
The proliferative subtype HGSOC exhibits the highest *MYBL2* expression across transcriptional subtypes. **(A,B)** Comparison of *MYBL2* expression across transcriptional subtypes (ANOVA with Tukey-HSD *post-test, p* < 0.01) with highest expression in the proliferative subtype. IR, Immunoreactive (*N* = 78); DIF, Differentiated (*N* = 67); MES, Mesenchymal (*N* = 71); PRO, Proliferative (*N* = 78). **(C)** High *MYBL2* expression is associated with a significantly worse prognosis in HGSOC (Figure 1) with a similar trend shown here in the proliferative subtype from TCGA data analysis. Data show Kaplan–Meier curves and Cox proportional hazard model.

To calculate the statistical significance of *MYBL2* differential gene expression, RT-qPCR data from at least 3 biological replicates was analyzed using two-sided Student's *t*-test JMP Pro 15 software. To investigate the effect of *MYBL2* expression on survival, the Ovarian Cancer RNA-seq data from TCGA (the “OV” cancer abbreviation) was analyzed. Gene expression data summarized as RSEM values were obtained using the TCGA2STAT R package v.1.2. The data were log2-transformed and analyzed using Kaplan–Meier curves and Cox proportional hazard model (Figure 1B). The modified approach from (14) was used to estimate the best gene expression cutoff that separates high/low expression subgroups with the most significantly differential survival. Only subgroups with >40 patients were considered, and the survival time was capped at 5 years. Scripts for performing TCGA survival analysis are available at https://github.com/mdozmorov/TCGAsurvival. Subtype-specific analysis (Figure 2) was performed on the TCGA-defined and annotated ovarian cancer transcriptional subtypes as previously described. Specifically, the proliferative subtype is defined by low *MUC1* and *MUC16* expression as well as high expression of *MCM2, PCNA, HMGA2*, and *SOX11* (6). Pearson correlation of MYBL2 with DREAM target genes (**Figure 4**) was performed using the rcorr function from the Hmisc v.4.4-1 R package. To investigate the effect of DREAM signature on survival (**Figure 6B**), we performed a single-sample GSEA analysis of each TCGA sample (the GSVA v.1.36.3 R package) to quantify the sample-specific enrichment score of DREAM gene expression. Samples were similarly separated into high/low enrichment of DREAM gene expression and analyzed for significant survival differences. The DREAM enrichment score was correlated with the *MYBL2* expression (**Figure 6A**). The curatedOvarianData R package v.1.26.0 (15) was used to evaluate *MYBL2* expression between different conditions, and two-sided *t*-test was used to assess significance (Figures 1C–E, Supplementary Figures 1A–D). Analyses were performed in R v.4.0.2 and visualized using the ggplot2 v.3.3.2 R package.

### Clinical Tumor Samples

The present study was carried forth under the provisions of the Declaration of Helsinki with approval by the Institutional Review Board of Virginia Commonwealth University (protocol code HM20009880 approved 04/28/2017 with associated protocol HM2471, further described as follows).

The Tissue and Data Analysis and Acquisition Core Laboratory (TDAAC) serves as a biorepository by acquiring and banking human cancers and adjacent normal tissues, as well as hematological samples for use in research. This is done through the aegis of the VCU IRB-approved “Tissue Acquisition System to Support Cancer Research” (TASSCR) protocol (protocol code HM2471), which can supply specimens to a biorepository supporting cancer research through acquisition of residual tumor and normal tissue samples along with informed consent from patients. Samples can thus be provided under an anonymous honest broker system. In addition, TDAAC collects tissue, hematopoietic, and other researcher-specific samples that support investigator-initiated, IRB-approved research projects or clinical trials. All frozen tissue specimens banked in TDAAC have a corresponding formalin-fixed, paraffin-embedded counterpart in the Department of Pathology archives. Patients who sign the TDAAC informed consent documentation agree to have their residual tissues and/or blood utilized for any research question, including genomic data and health information for translational research.

In the present study, 49 of the 52 banked frozen tissue specimens had concurrent formalin-fixed, paraffin-embedded surgical oophorectomy specimens available. Histologic sub classification of HGSOC according to the protocol outlined by Murakami et al. (17) was reviewed by two board-certified anatomic pathologists with expertise in gynecologic pathology. H&E stained slides were independently reviewed and categorized as mesenchymal transition (MT) if there was a complex, labyrinthine pattern with >10% desmoplastic reaction, immunoreactive pattern (IR) when the tumor had rounded contours and associated tumor–infiltrative lymphocytes, papilloglandular (PG) when there was papillary growth pattern or solid and proliferative (SP) when there was solid architecture without significant desmoplastic reaction (17). Both pathologists were blinded to the clinical data and the research question and categorization were finalized when a consensus was reached.

To evaluate p53 expression in these samples a tissue microarray was created from representative formalin-fixed paraffin embedded tissue blocks of the tumor. Immunohistochemical staining was performed with p53 (Dako Omnis p53 protein clone DO-7) and interpreted by 3 gynecologic pathologists for the presence of aberrant vs. wild-type expression. Aberrant expression was characterized as a complete absence of staining (Null) or diffuse staining (Positive). Normal or non-aberrant expression was characterized by heterogeneous staining (wild-type).

## Results

### MYBL2 Is Highly Expressed in the Majority of HGSOC Cases and Is Associated With Poor Overall Survival

We first sought to characterize *MYBL2* expression in HGSOC. Compared with other disease sites in the available PanCan TCGA studies, HGSOC had the third highest alteration frequency, surpassing that of invasive breast carcinoma, for which *MYBL2* (encoding B-Myb) carries a clinically significant predictive value (Figure 1A) (4, 5). Our previous work demonstrated that *MYBL2* copy number alterations are correlated with mRNA expression (7). Similar to invasive breast carcinoma, high expression of *MYBL2* was significantly associated with poorer overall survival in TCGA cases (Figure 1B) (16).

*MYBL2* was expressed at significantly higher levels in primary untreated ovarian carcinoma as compared with borderline ovarian surface epithelial-stromal tumor (Figure 1C) and ranked as the fourth most differentially upregulated gene among an independent data set (*t*-test 18.076, *p* = 5.99E-31, fold change 2.555, data not shown) (6, 16, 18). Similarly, *MYBL2* exhibited higher expression in HGSOC tumor samples than healthy ovarian surface epithelium controls (Supplementary Figures 1A,B) (19, 20). *MYBL2* was more highly expressed in high grade (Figure 1D, Supplementary Figure 1C) and late stage tumors (Figure 1E, Supplementary Figure 1D) (16). To validate these findings, we performed RT-qPCR analysis of *MYBL2* expression on our independently collected set of clinical HGSOC tumor samples. This retrospective investigation utilized tissue banked surgical pathology and cytology samples that were taken from 57 HGSOC lesions collected between November 2000, and April 2017. Demographic and disease data were obtained by chart review by an investigator blinded to the primary research question. Clinical data were available for 52 of the 57 analyzed tumor tissue samples. The clinical characteristics of these patients are described in Table 1. A panel of housekeeping control genes (18S, actin, GAPDH) were directly compared across 3 samples to determine which yields the most consistent results across tumor samples (data not shown) (21, 22). This led us to proceed with 18S ribosomal RNA as our housekeeping control.

**Table 1:** Clinical characteristics of HGSOC primary tumor lesions.

**Variable**	**Category**	**Proportion**	**%**
Age (years)	≤65	39/52	75
	≥65	13/52	25
Stage	2	2/52	4
	3	41/52	79
	4	9/52	17
Optimal debulking	Yes	22/45	49
	No	23/45	51
Adjuvant chemotherapy regimen	None	5/45	11
	Carboplatin+taxane	39/45	87
	Single agent carboplatin	1/45	2
BRCA status	No identified mutation	9/12	75
	BRCA1 mutation	0/12	0
	BRCA2 mutation	1/12	8
	Other pathogenic mutation	2/12	17
Recurrence	No	7/34	21
	Yes	27/34	79
Platinum sensitivity	No (≤6 months between chemotherapy completion and recurrence)	12/33	36
	Yes (≥6 months between chemotherapy completion and recurrence)	21/33	64

*Demographic and disease data were obtained by chart review by an investigator blinded to the primary research question. N = 52 unless otherwise noted*.

TCGA analysis showed genetic alterations resulting in aberrantly high expression of *MYBL2* in ~55% of HGSOC cases. In our study population, 51% of samples had *MYBL2* expression levels greater than that of two adjacent normal-like tissue samples (Supplementary Figure 1E). *MYBL2* expression did not impact overall survival in our patient cohort which may, in part, be attributable to the modest sample size (Supplementary Figure 1F). Since multiple independent sets of cDNA were prepared, *MYBL2* expression was also used compared across batches of RNA to validate reproducibility (Supplementary Figure 2).

We observed variable expression across the HGSOC cases (Supplementary Figure 1E). We compared *MYBL2* expression across transcriptional subtypes with TCGA and found the highest expression in the proliferative subtype (Figure 2A). *MYBL2* was significantly highly expressed in the proliferative subtype as compared with the differentiated subtype (*p* = 1.51E-8), which had the lowest expression. The immunoreactive subtype, with the second highest *MYBL2* expression, also exhibited significantly higher *MYBL2* expression as compared with the differentiated subtype (*p* = 1.208E-7) but did not differ from that in the proliferative subtype (*p* = 0.98; Figure 2B). Interestingly, high *MYBL2* expression was associated with poor overall survival in the proliferative subtype (log-rank *p* = 0.017; Figure 2C), similar to the effect observed in HGSOC collectively (Figure 1B).

**Figure 3:**
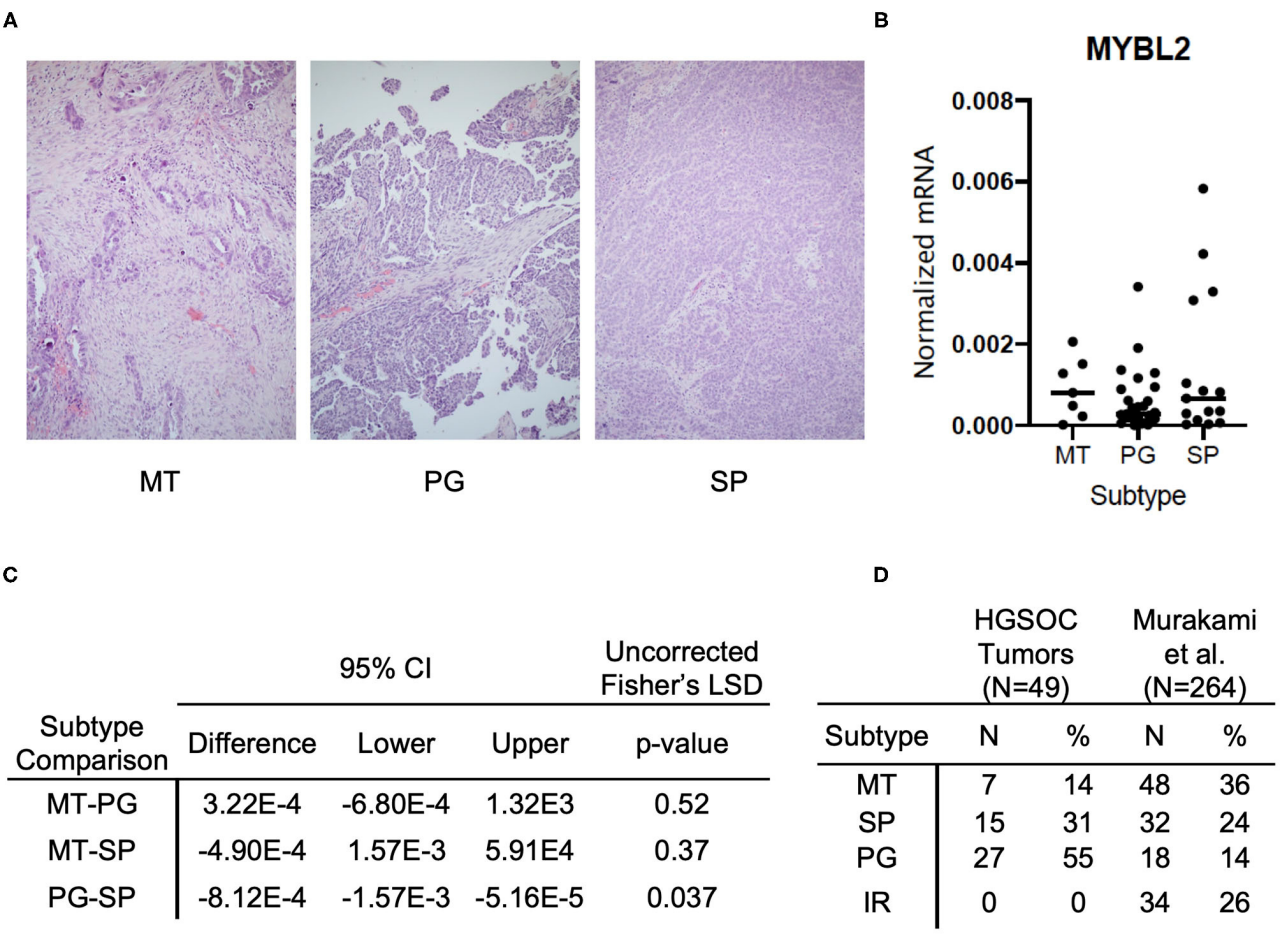
*MYBL2* expression varies by histological subtype and is associated with morphological evidence of cell proliferation. **(A)** Representative H&E sections of respective histological subtypes at 4× magnification. (MT) Mesenchymal Transition subtype characterized by prominent desmoplastic reaction (*N* = 7). (PG) Papilloglandular subtype with predominance of papillary architecture (*N* = 27). (SP) Solid and Proliferative subtype characterized by solid tumor nests with rounded contours (*N* = 15). The immune reactive (IR) subtype, defined by rounded contours and infiltrative lymphocytes, was absent in our samples. **(B)** Graph shows comparison of *MYBL2* expression across HGSOC primary tumor samples (*N* = 49) grouped by histological subtype **(C)** Analysis of differential *MYBL2* expression across histological subtypes. Uncorrected Fisher's least significant difference, *p* < 0.05. **(D)** Table showing distribution of samples across subtypes alongside those in the original study by Murakami et al.

To further understand *MYBL2* expression by subtype, we applied histological classification of tumor samples. Tissue classified as solid and proliferative subtype exhibited significantly higher *MYBL2* expression as compared with the papilloglandular subtype (Figures 3A–C). The solid and proliferative subtype was previously not found to have a significant overlap in gene expression signature with the TCGA transcriptional subtypes and had no impact on overall survival (17, 23). *MYBL2* was highly expressed in the proliferative transcriptional subtype (Figures 2A,B) as well as tumors with morphological features consistent with cell proliferation (solid and proliferative histological subtype) (Figures 3B,C). Of note, there were no cases of the immune reactive histological subtype among our 52 clinical HGSOC tumor samples (Figure 3D). P53 expression by immunohistochemical staining confirmed aberrant expression in 40 of 49 samples. Of the 9 tumors showing wild-type expression, two had morphologic features consistent with high-grade endometrioid and clear cell carcinoma.

Overall, we have shown that *MYBL2* is highly expressed in HGSOC as would be predicted by its genetic alterations and have validated this finding in 3 independent data sets. We determined that *MYBL2* is most highly expressed in the proliferative transcriptional subtype of TCGA HGSOC as well as the solid and proliferative histological subtype. Furthermore, high *MYBL2* expression is associated with poor overall survival in HGSOC cases collectively as well as in the proliferative transcriptional subtype. *MYBL2*'s high expression in both the proliferative transcriptional and solid and proliferative histological subtypes is consistent with B-Myb's known role in MMB complex formation and cell cycle progression. Given these findings, we next sought to further evaluate the mechanism by which B-Myb alters cell cycle regulation in HGSOC.

### High B-Myb Expression Is Associated With the Expression of DREAM Target Genes

**Figure 4:**
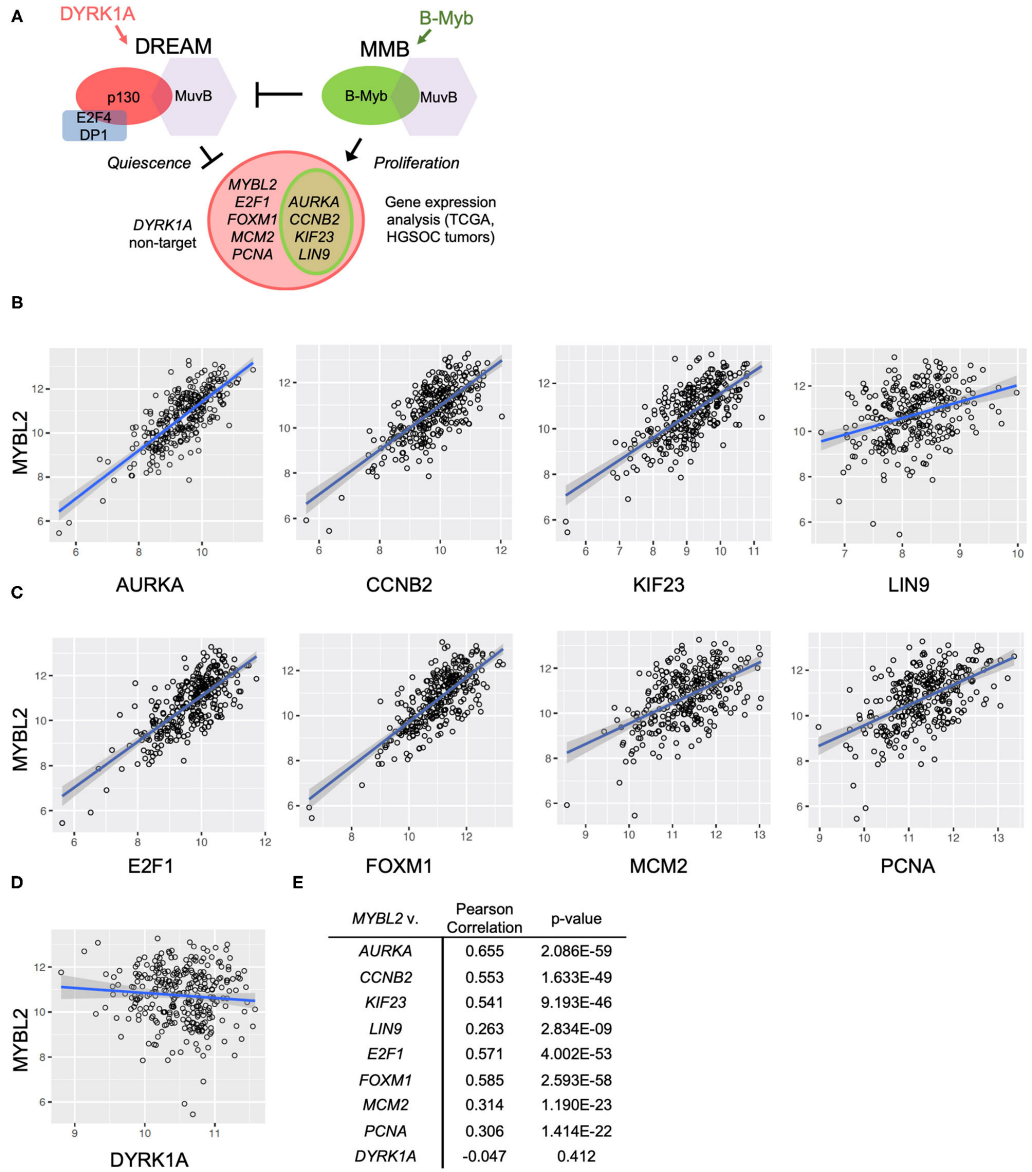
*MYBL2* expression correlates with DREAM target gene expression in the TCGA data set. **(A)** Model for B-Myb-mediated DREAM disruption. High B-Myb levels promote DREAM disassembly and, in turn, expression of genes normally repressed by intact DREAM. Genes of interest are shown along with control non-DREAM target gene, *DYRK1A*. Expression of DREAM target genes serves as a functional readout for DREAM formation. **(B,C)** HGSOC TCGA gene expression data relating *MYBL2* expression to DREAM **(B)** and DREAM/MMB **(C)** target genes as well as non-DREAM/MMB target control *DYRK1A* **(D)** (*N* = 303). **(E)** Results of TCGA Pearson correlations analyses and associated *p*-values.

High B-Myb expression disrupts repressive DREAM complex formation in human cell lines. Our previous analysis of TCGA data supported our cellular model by showing that *MYBL2* undergoes gene copy number gain in the majority of HGSOC tumor samples and, in turn, is associated with increased expression of DREAM and MMB target genes (7). We sought to validate these findings with clinical specimens and further characterize DREAM as well as MMB functional status in HGSOC tumors. To this end, we assessed the expression of DREAM and MMB controlled genes as a functional readout for the status of these complexes (Figure 4A).

The DREAM and MMB target genes for validation of our model were selected based on criteria of high differential gene expression in our previous analysis, established cell cycle role, and clinical interest. Using existing databases for DREAM and MMB target genes, we verified that our selections were indeed annotated targets (24). These steps collectively yielded the following genes of interest: *MYBL2, PCNA, MCM2, AURKA* (25), *KIF23* (26), *CCNB2* (27), *LIN9* (28), *E2F1* (29), and *FOXM1* (30–35). Relevance of these genes in HGSOC pathogenesis, prognosis and treatment response provides the rationale for the studies described below.

*FOXM1* was included in our study due to previously reported robust upregulation and potential prognostic role in HGSOC (6, 30, 35, 36). Aurora kinase A (*AURKA*) was also included since it was recently shown to modulate epithelial ovarian cancer cell adhesion and migration, in turn, promoting cancer cell dissemination. *KIF23*, a mitotic kinesin, was previously characterized as an MMB target gene important for tumorigenesis in an oncogenic K-RAS-driven mouse model of lung adenocarcinoma. Cyclin B2 (*CCNB2*) was further investigated since it appeared near the top of the list of differentially expressed genes in the presence of high B-Myb level, and transgenic mice expressing high levels of Cyclin B2 level are prone to tumor development (7). Another gene of interest, *LIN9*, is highly expressed in triple negative breast cancer and is associated with poor outcomes in this disease (28). Finally, *E2F1* was included given its well-known role as a mediator of cell growth and its contribution to transcription of the matrix metalloproteases MMP2 and MMP9 that promote migration and invasion (29). We also included the *DYRK1A* gene because of the key role its product plays in the DREAM assembly. DYRK1A kinase phosphorylates MuvB component LIN52, a required step for DREAM complex assembly and factor in the mechanism of B-Myb-mediated DREAM disruption (7, 9). Although DYRK1A is a key component of the DREAM regulatory pathway, it is not subject to transcriptional regulation by MuvB-containing complexes making it a suitable experimental control (9). However, similarly to high B-Myb expression, the *DYRK1A* gene copy number loss, observed in 38% of HGSOC cases, could be another mechanism leading to decreased DREAM assembly.

To test the hypothesis that high expression of B-Myb is associated with decreased DREAM complex formation and a correspondent upregulation of DREAM targets, we compared the expression of *MYBL2* and a panel of DREAM target genes described above, using the TCGA HGSOC data set (6). We simultaneously tested the impact of high *MYBL2* expression on DREAM formation in our own set of patient-derived tumor samples. Additionally, as noted in Figure 4A, we included a number of genes that are regulated by both DREAM and MMB (*AURKA, CCNB2, KIF23, LIN9*) for which we anticipated similar findings as genes targeted only by DREAM.

**Figure 5:**
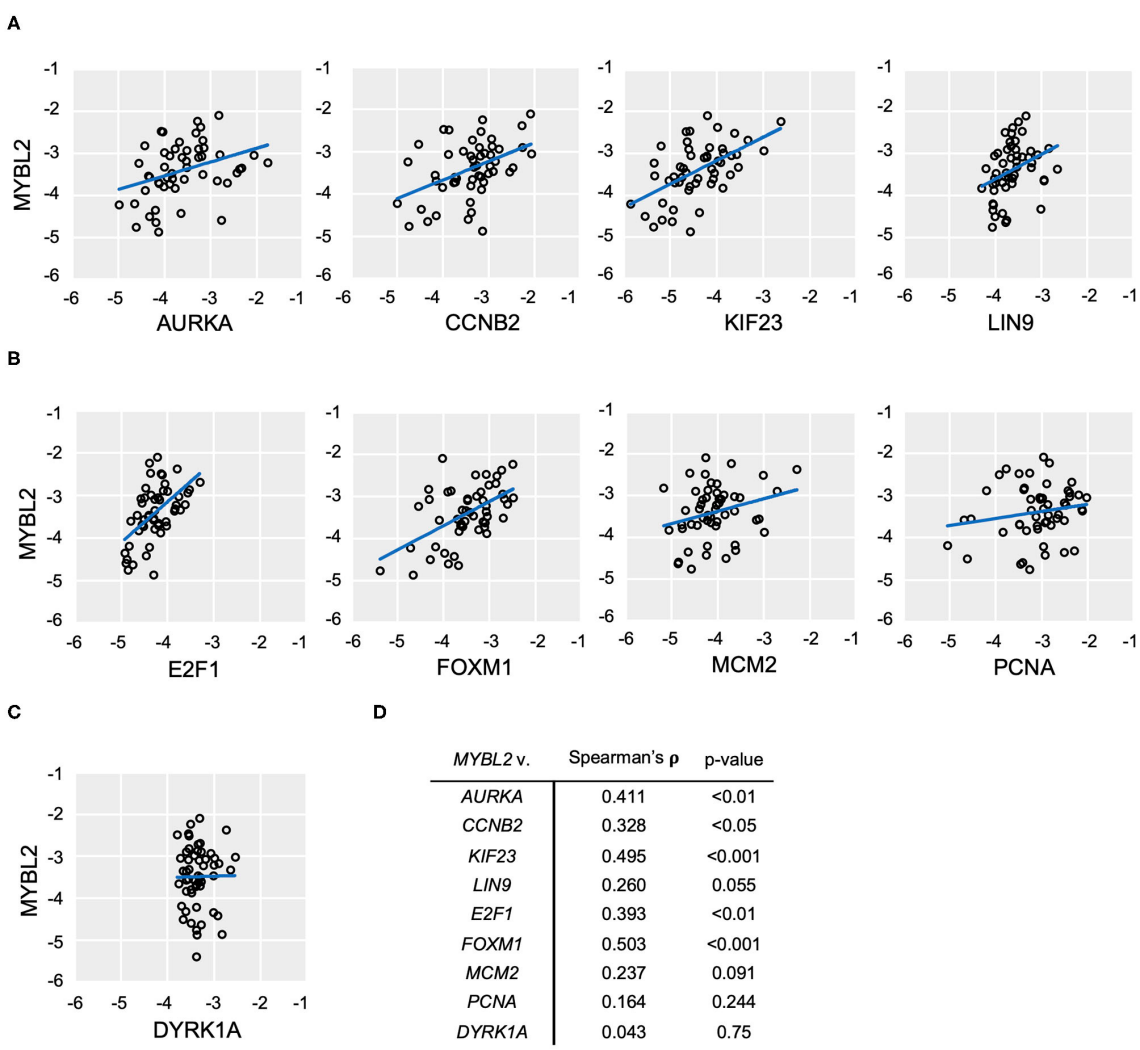
*MYBL2* expression correlates with DREAM target gene expression. **(A–C)** RT-qPCR analysis of human ovarian tumor surgical sections (*N* = 52). As in Figure 4, expression of DREAM **(A)** and DREAM/MMB **(B)** target genes is shown relative to that of *MYBL2* as well as non-DREAM/MMB target, *DYRK1A*. Plots show log_10_ mRNA expression relative to 18S ribosomal RNA housekeeping control. **(C)** Non-parametric Spearman Rank Correlations were used for analysis. **(D)** Results of non-parametric Spearman's rho and associated *p*-values.

In support of B-Myb-mediated DREAM disruption, Pearson correlation analysis revealed positive and significant correlations between expression of *MYBL2* and each of the representative DREAM target genes in the TCGA data set (Figure 4B). The same analysis produced similar findings in the DREAM/MMB target genes (Figure 4C, normalized expression values for individual genes in Supplementary Figure 3). The expression of non-DREAM target *DYRK1A* did not significantly correlate with *MYBL2* expression (*r* = −0.047, *p* = 0.41; Figures 4D,E). RT-qPCR analysis of DREAM target genes from our patient-derived HGSOC tumor samples produced positive and significant correlations between *MYBL2* and all of the selected DREAM and DREAM/MMB target genes, with the exception of LIN9: *LIN9* (ρ = 0.259, *p* = 0.055), *AURKA* (ρ = 0.411, *p* < 0.01), *KIF23* (ρ = 0.495, *p* < 0.001), *CCNB2* (ρ = 0.328, *p* < 0.05), *E2F1* (ρ = 0.392, *p* < 0.01), and *FOXM1* (ρ = 0.503, *p* < 0.001; Figures 5A,B). Whereas, *LIN9* and *MYBL2* expression were not correlative in our patient samples, *LIN9* did significantly correlate with *MYBL2* expression in the TCGA data (*r* = 0.263, *p* = 2.834E-09), albeit weakly relative to the rest of the genes (Figures 4B, 5A). Similar to our previous analysis (Figure 4D), *DYRK1A* expression did not significantly correlate with that of *MYBL2* (*p* = 0.043, *p* = 0.75; Figures 5C,D). Collectively, these data support our model, as evidenced by the expression of DREAM and DREAM/MMB target genes positively correlating with *MYBL2* expression. These data suggest a possible *MYBL2* amplification gene expression signature characterized by de-repression of DREAM target promoters.

**Figure 6:**
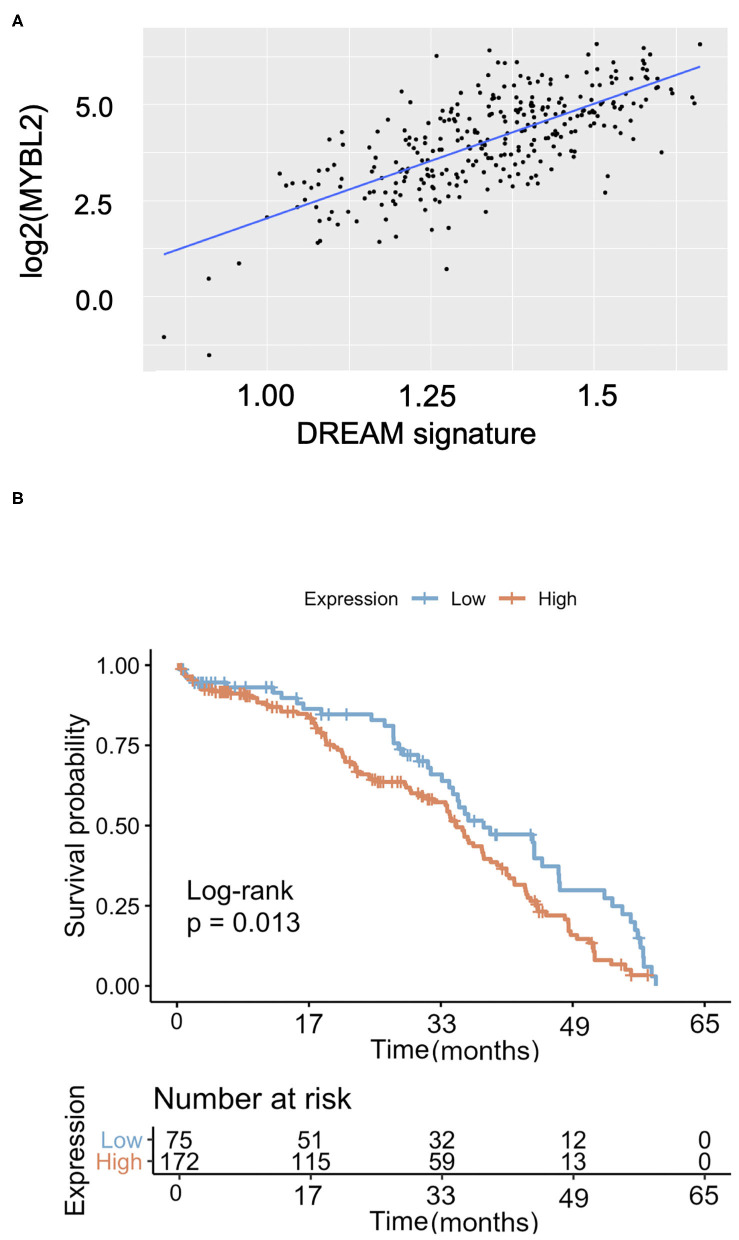
The DREAM transcriptional signature in HGSOC. **(A)** *MYBL2* expression is positively and significantly correlated with global expression of annotated DREAM target genes [as described in Fischer et al. (24) NAR 2016] in TCGA HGSOC samples (*N* = 303, Pearson correlation = 0.6, *p* < 0.0001). **(B)** Kaplan Meier survival curve according to DREAM transcriptional signature expression (*N* = 303).

Since B-Myb expression correlated with a subset of clinically relevant DREAM target genes, we then investigated whether this relationship holds true across all DREAM target genes. Using a recently updated list of annotated DREAM target genes, we performed Pearson correlation analysis of the sample-specific enrichment scores in DREAM target genes (single-cell GSEA, see Methods) with *MYBL2* expression in TCGA data (24). Consistent with our single gene analyses, *MYBL2* expression was positively and significantly correlated with the collective DREAM expression signature (*N* = 303, *r* = 0.6, *p* < 0.01), suggesting that B-Myb expression may serve as a surrogate marker for DREAM status in HGSOC (Figure 6A). Expression of the DREAM signature was also associated with decreased overall survival (log-rank *p* = 0.013; Figure 6B). Taken together, these findings argue that there is a mechanistic relationship between B-Myb and DREAM. Each may serve as prognostic markers in HGSOC and B-Myb may have prognostic significance independent of its cell cycle effects.

## Discussion

We demonstrated that increased expression of selected cell cycle genes correlates with expression of DREAM and MMB-regulated genes in HGSOC tissue. High expression of *MYBL2* is associated with deregulated cell cycle gene expression programs in HGSOC, suggesting that it may play an important role in the pathogenesis and clinical outcomes of patients. Though the mechanism for which B-Myb confers a poor prognosis is not fully understood, high B-Myb expression contributes to DREAM disruption (7). This mechanism, in turn, suggests that the status of DREAM assembly and expression of DREAM-regulated genes might play a prognostic role in HGSOC as well. Interesting, both high B-Myb expression and expression of DREAM target genes were positively correlated and associated with poor overall survival (Figures 1B, 6B). These findings are counter to the role of the DREAM complex in maintaining cellular dormancy and, in turn, chemoresistance (12). B-Myb and DREAM status may have independent prognostic implications despite their mechanistic relationship at the cellular level. However, a positive correlation between *MYBL2* and DREAM target gene expression despite variable degrees of *MYBL2* expression was noted. A limitation to this study is the direct comparison between DREAM target gene expression and survival in our independent patient cohort. Larger studies would clarify the clinical prognostic value of the DREAM-regulated gene expression. This study is also operating on the premise that gene expression likely results in translation of functional protein, but does not validate this rationale with protein level studies. Additionally, it would be valuable to compare gene expression between HGSOC tumors and healthy control fallopian tube epithelial cells. This would allow improved definitions of “cutoff” points for “high” gene expression (21). Further correlation with p53 mutational status is also needed to explain the presence of wild-type expression seen in seven remaining tumors of our tumor samples. The presence of a truncating mutation, over interpretation of cytoplasmic staining, the presence of a mixed carcinoma, and tumor heterogeneity are possible explanations for a normal expression pattern.

We found that *MYBL2* is upregulated in the proliferative subtype and in tumor tissue with histologic evidence of proliferation (Figures 2, 3). The lack of immune reactive histological subtype samples among our data set suggest potential demographic differences across patient populations or may be a result of limited number of available tissue samples. The proliferative subtype is genetically defined by high expression of proliferation markers, *MCM2* and *PCNA* (6). Both of these markers are DREAM target genes and significantly correlate with *MYBL2* expression (Figures 4C, 5B) (24). Their high expression, and co-expression with other DREAM targets, is consistent with a phenotype of DREAM disruption. Our findings may alternatively reflect a greater proportion of dividing cells in these samples compared with other subtypes. In this case, dividing cells will physiologically have less DREAM formation, leading to de-repression of *MYBL2* (which is itself a DREAM target gene) and, in turn, more DREAM disruption. A positive feedback loop might be another factor driving the HGSOC proliferation alongside other mechanisms that increase B-Myb expression (such as genomic amplification) and supported by our subtype analysis (Figure 2) (7).

The status of DREAM assembly is of interest in relation to the FoxM1 transcription factor network, which is activated in >84% of HGSOC cases (6). Previous studies support FoxM1 upregulation through genomic amplification of *FOXM1*, inhibition of p53 and pRb, and E2F1 activation. These previous studies, however, did not assess DREAM status (35). Our results support the model of DREAM disruption by high *MYBL2* expression as another potential mechanism driving FoxM1 activity. *MYBL2* and *FOXM1* expression are upregulated in many p53 mutant cancers such as HGSOC (37, 38). Although the role of B-Myb and FoxM1 upregulation in cancer progression is not fully understood, high expression of these factors can contribute to abnormal mitosis and chromosomal instability (39). Furthermore, *FOXM1* is also part of chromosomal instability transcriptional signatures (CIN70 and CIN25), characteristic of aneuploid tumors (40). Decreased B-Myb level results in lower expression of G2/M phase-expressed genes and mitotic arrest. Similarly, FoxM1 depletion results in delayed mitotic entry as well as defective mitosis and cytokinesis (41). The importance of FoxM1-MuvB to lung and breast cancer pathogenesis has been described, but further studies in HGSOC are needed to define the molecular mechanisms by which these transcription factors exert their unfavorable effects and assess the routes of potential therapeutic development (26, 42). This is particularly relevant in the context of response to chemotherapy as FoxM1 upregulates the expression of genes involved in DNA damage and repair pathways, contributing to treatment resistance (31–33).

Along with *FOXM1*, DREAM status also influences the expression of several other clinically relevant genes: *AURKA* (Aurora kinase A), *CCNB2* (Cyclin B2), *KIF23*, and *LIN9*. The therapeutic potential of Aurora kinase A inhibitor-taxane combination treatment is being explored through orthotopic xenograft models (25). This approach might indirectly influence the actions of Cyclin B2. One proposed mechanism for Cyclin B2's tumorigenic properties is by promoting aneuploidy through stimulating Polo-like kinase 1 activation in an Aurora kinase A-dependent manner (27). Depletion of *KIF23* is also suggested as another potential cancer therapeutic approach for lung adenocarcinoma (26). Finally, may provide the opportunity for more targeted treatment since the function of an experimental class of mitotically-active drugs, bromodomain and extraterminal protein inhibitors (BETi), is linked with *LIN9* expression, suggesting that patients with high *LIN9* expression may be more responsive to this treatment (28).

Our findings collectively suggest potential therapeutic angles for restoring cell cycle control in HGSOC. Though DREAM is implicated in harboring disease recurrence (12), inhibition of B-Myb and, in turn, restoration of DREAM assembly by CDK inhibition may of therapeutic value for the proliferative subtype of HGSOC as well as in cases of *FOXM1* (12% of cases) or *MYBL2* (55% of cases) gains (35). Increased DREAM formation may curb the pathogenic mechanisms enacted by FoxM1 (6, 35). This strategy might have secondary effects of repressing DNA damage repair genes, sensitizing cells to PARP inhibitors, (33) and enhancing responses to paclitaxel and platinum agents in chemotherapy-resistant disease (34).

In conclusion, we propose a mechanism by which high *MYBL2* expression is associated with poor prognosis through DREAM disruption in HGSOC patients. Targeting and inhibiting B-Myb may be a viable treatment option for selected patients. Furthermore, given that B-Myb expression levels are often increased in HGSOC, and its prominent cell cycle effects, it is worthwhile to investigate B-Myb's potential as a predictive or functional biomarker in HGSOC transcriptional subtypes.

## Data Availability Statement

Publicly available datasets were analyzed in this study. This data can be found at: https://www.cancer.gov/about-nci/organization/ccg/research/structural-genomics/tcga.

## Ethics Statement

The studies involving human participants were reviewed and approved by Virginia Commonwealth University IRB. The patients/participants provided their written informed consent to participate in this study.

## Author Contributions

AI, ST, and LL designed the study. AI and LL wrote the manuscript. AI performed experiments and analyzed data. AI, LR, JC, SM, and MD analyzed gene expression and survival data. AI, SM, MD, and LL prepared figures. SS and RP reviewed histological samples. ST, MD, SS, and RP edited the manuscript. All authors contributed to the article and approved the submitted version.

## Conflict of Interest

The authors declare that the research was conducted in the absence of any commercial or financial relationships that could be construed as a potential conflict of interest.
