# Supplementary material for: Oncogenic B-Myb Is Associated With Deregulation of the DREAM-Mediated Cell Cycle Gene Expression Program in High Grade Serous Ovarian Carcinoma Clinical Tumor Samples
Source: Front Oncol. 2021 Mar 4;11:637193. doi: 10.3389/fonc.2021.637193 (PMC7969987; doi:10.3389/fonc.2021.637193)
Supplement: Supplementary file 1 [file Data_Sheet_1.PDF]

Iness et al.,
Figure S1

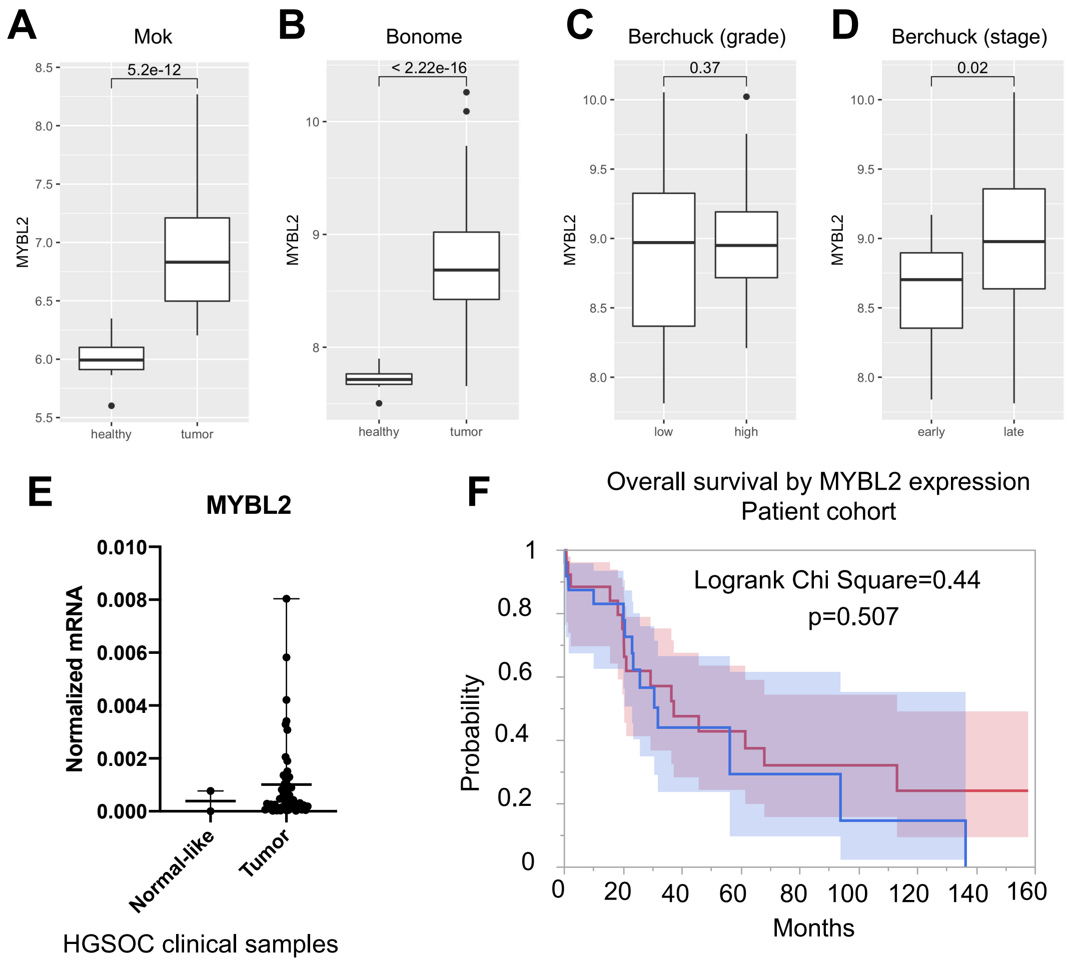

Expression	Median time	Lower 95%	Upper 95%	Number failed	Number censored	SE
Low	37.18	20.22	113.03	16	11	9.38
High	31.78	20.58	93.83	14	11	12.66
			Combined:	30	22	8.36

Figure S1. *MYBL2* is highly expressed in HGSOC. (A) Published gene expression dataset GSE18520 (1) was probed for *MYBL2* expression in healthy ovarian surface epithelium (N=10) compared with HGSOC tumor samples (N=53). **(B)** As in panel A, *MYBL2* expression from GSE26712 (2) comparison between healthy ovarian surface epithelium (N=10) and HGSOC tumor samples (N=185). **(C, D)** Study by Berchuck et al., 2005 (3) was analyzed for MYBL2 expression in serous ovarian carcinoma by high grade (N=25) versus low grade (N=37) **(C)** and comparison by stage (early N=11 versus late N=185) **(D)**. **(E)** *MYBL2* mRNA expression in 52 HGSOC clinical tumor samples normalized to 18S housekeeping gene as compared with *MYBL2* expression values in two adjacent normal-like tissue samples. **(F)** Kaplan-Meier analysis of overall survival of patient cohort corresponding to expression data in (E). “Low” and “High” expression were designated by formation of two quantiles.

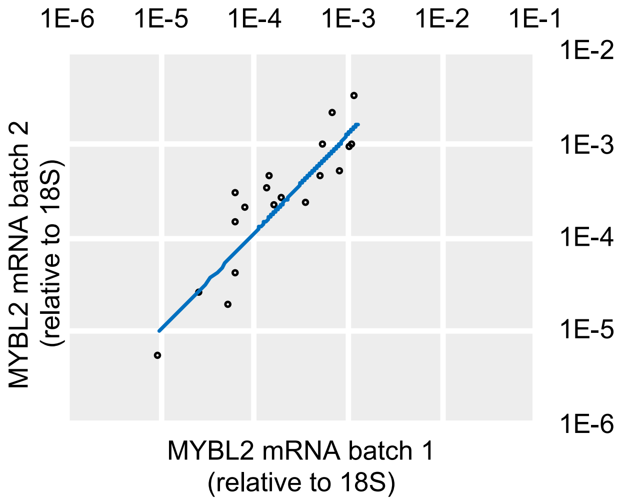

Spearman's ρ	p-value
0.91	<0.0001

Figure S2. Gene expression remained consistent across multiple mRNA batch preparations. Comparison of *MYBL2* gene expression (normalized to 18S ribosomal RNA expression) across two independent batches of mRNA samples from the same tumors (N=19). Spearman's rho = 0.913, $p < 0.0001$.

Iness et al., Figure S3

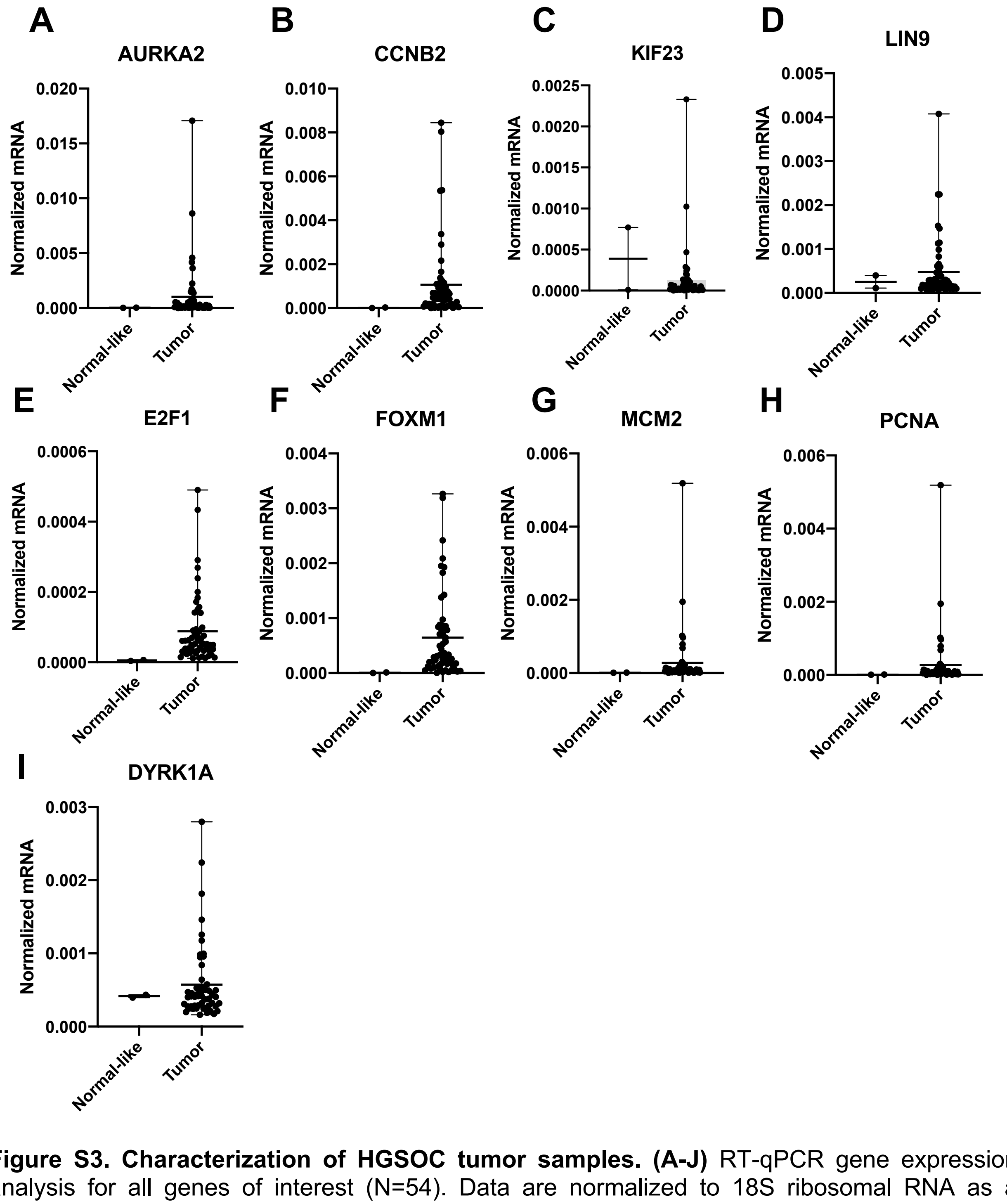

Figure S3. Characterization of HGSOC tumor samples. (A-J) RT-qPCR gene expression analysis for all genes of interest (N=54). Data are normalized to 18S ribosomal RNA as a housekeeping gene control.

References

1. Mok SC, Bonome T, Vathipadiekal V, Bell A, Johnson ME, Park D-C, et al. A gene signature predictive for outcome in advanced ovarian cancer identifies a survival factor: microfibril-associated glycoprotein 2. Cancer cell. 2009;16(6):521-32.
2. Bonome T, Levine DA, Shih J, Randonovich M, Pise-Masison CA, Bogomolniy F, et al. A gene signature predicting for survival in suboptimally debulked patients with ovarian cancer. Cancer research. 2008;68(13):5478-86.
3. Berchuck A, Iversen ES, Lancaster JM, Pittman J, Luo J, Lee P, et al. Patterns of gene expression that characterize long-term survival in advanced stage serous ovarian cancers. Clinical cancer research. 2005;11(10):3686-96.
